# Non-binding relationship between visual features

**DOI:** 10.3389/fnhum.2014.00749

**Published:** 2014-10-08

**Authors:** Dragan Rangelov, Semir Zeki

**Affiliations:** ^1^Psychology Department, Ludwig-Maximilians-Universität MünchenMunich, Germany; ^2^Wellcome Laboratory of Neurobiology, University College LondonLondon, UK

**Keywords:** visual perception, object binding, perceptual asynchrony, focal attention, multinomial models

## Abstract

The answer as to how visual attributes processed in different brain loci at different speeds are bound together to give us our unitary experience of the visual world remains unknown. In this study we investigated whether bound representations arise, as commonly assumed, through physiological interactions between cells in the visual areas. In a focal attentional task in which correct responses from either bound or unbound representations were possible, participants discriminated the color or orientation of briefly presented single bars. On the assumption that representations of the two attributes are bound, the accuracy of reporting the color and orientation should co-vary. By contrast, if the attributes are not mandatorily bound, the accuracy of reporting the two attributes should be independent. The results of our psychophysical studies reported here supported the latter, non-binding, relationship between visual features, suggesting that binding does not necessarily occur even under focal attention. We propose a task-contingent binding mechanism, postulating that binding occurs at late, post-perceptual (PP), stages through the intervention of memory.

## Introduction

The brain consists of many visual areas which differ in their specializations for different visual attributes as well as in their temporal dynamics (Zeki, 1978; Livingstone and Hubel, 1984, 1988; Hubel and Livingstone, 1985; Shipp and Zeki, 1985; DeYoe and Van Essen, 1988; Zeki et al., 1991). The latter is reflected in a perceptual asynchrony, with some attributes of the visual scene such as color being perceived before other attributes such as motion or orientation (Moutoussis and Zeki, 1997a,b; Viviani and Aymoz, 2001; Holcombe and Cavanagh, 2008). How attributes processed in different locations and at different times are bound together to give us our unitary experience of the visual world remains a matter of debate (Treisman, 1999; Valdes-Sosa et al., 2000; Wheeler and Treisman, 2002; Wylie et al., 2004). The absence of a consensus about how binding occurs has encouraged us to entertain a more radical possibility, namely that binding does not occur by interaction between single cells at the level of sensory cortices or, if it does, it is perceptually ineffective.

Even if there are pluripotent cells, which respond to many or all the different attributes of the visual scene such as color, form and orientation, as some have suggested (e.g., Leventhal et al., 1995), perceptual asynchrony creates a problem that remains un-addressed, namely what mechanism allows cells to integrate and bind signals that are processed at different speeds and therefore perceived with different latencies? Here, we posit that different attributes are processed independently and are not bound physiologically but only *experienced* as being bound through the intervention of post-perceptual (PP) processes. A PP binding mechanism would be strongly supported by a demonstration of significant differences between tasks that do and those that do not require bound stimulus representations. Having to bind different stimulus attributes slows down stimulus processing and decreases response accuracy, summarized in the term “binding costs” (Treisman and Gelade, 1980; Treisman, 1982; Wolfe, 1994, 2007; Bodelón et al., 2007). Binding costs are inconsistent with early perceptual binding, because should bound representations arise from cells in visual sensory cortex which bind all features, there should be no differences between conditions and therefore no binding costs.

To explain binding costs, Treisman and Gelade (1980), Treisman (1999) and Wheeler and Treisman (2002) proposed the Feature Integration Theory (FIT), which assumes that *focal attention mediates* binding processes so that only the attributes of attended stimuli are bound while the attributes of stimuli that are not focally attended are processed independently (Bundesen et al., 2003; Kyllingsbæk and Bundesen, 2007). This hypothesis has, however, been challenged by studies in which two stimuli, the target and distractor, are presented simultaneously at the attended location. Using such stimuli, numerous studies have shown a superior processing of *all* attributes of the target, those that are task-relevant as well as those that are not compared to the distractor stimulus (Duncan, 1984, 1985; O'Craven et al., 1999; Driver et al., 2001; Holcombe and Cavanagh, 2001, 2008). Later studies which controlled for stimulus related confounds reported similar results: attending to the color of a moving surface necessarily involves processing of its motion, despite the fact that motion is task irrelevant (e.g., Valdes-Sosa et al., 1998, 2000; Rodrìguez et al., 2002; López et al., 2004; Katzner et al., 2009). These findings were taken as empirical evidence that focal attention operates at the level of already bound objects, implying that binding occurs very early and before focal attention.

In summary, despite substantial differences between different accounts, both the attention-mediated and the pre-attentive binding mechanisms imply that, once attention is allocated, different attributes of a stimulus are bound. Thus, a critical test for existing theories of binding is whether or not different attributes of attended objects are mandatorily bound. Using different variants of a visual search paradigm, previous studies reported evidence for statistical independence of response accuracy for two attributes of the *attended* item, suggesting that even attended items are not processed in a bound way (Isenberg et al., 1990) thus strengthening the suspicion that attention and binding are independent of each other.

However, it is possible to account for these results in other ways. Since in visual search tasks it is uncertain which stimulus is the target, it is possible that attention selects *several* items whose features are then erroneously bound. Random-sampling of features which are all selected by a broad attentional spotlight might offer a mechanism through which attention-mediated binding could still yield statistical independence of different stimulus attributes (Vul and Rich, 2010). It could thus in principle reconcile mandatory binding and the evidence showing independence between reporting attributes of the attended object. Random-sampling of attended and encoded features, which leads to random binding seems to be supported by recent studies. If features from attended locations are indeed randomly sampled, then erroneous reports would reflect misbinding processes, i.e., both features are correctly reported, but these features belong to *different* objects. This prediction was supported by showing that assuming misbinding processes is necessary to account for response variability in reporting colors (Bays et al., 2009).

**Figure 1:**
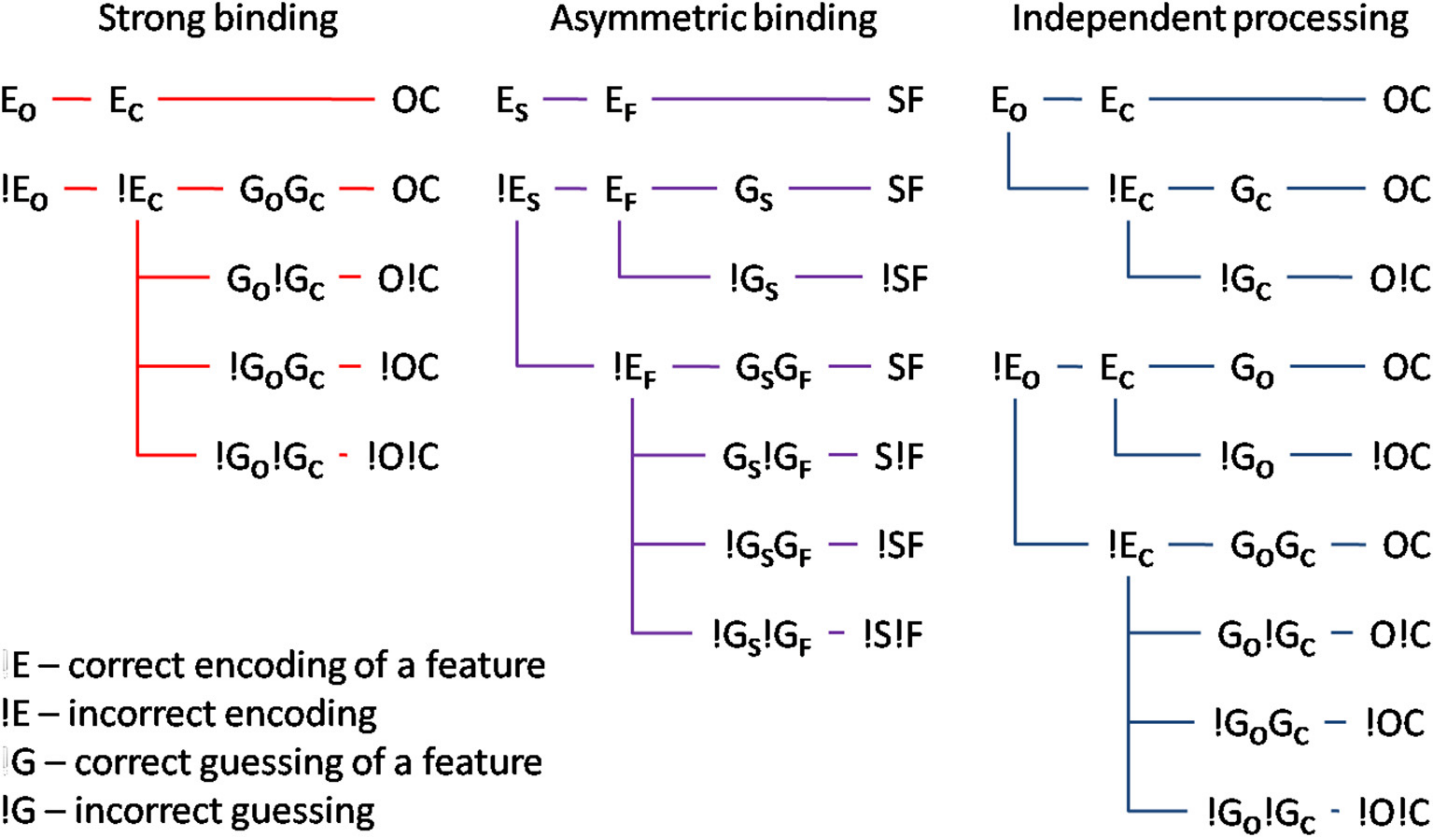
**Illustration of different models: (i) strong binding—red, (ii) asymmetric binding—violet, and (iii) independent processing—blue**. The stimulus attributes could have been correctly encoded (E_o_ and E_c_ for encoding of orientation and color respectively). If the encoding failed (!E), random guessing processes take place (G_o_ and G_c_ for correct guesses and !G for respective incorrect guesses). Different combinations of encoding and guessing yield four possible response types: (i) both correct responses—OC, (ii) correct orientation, wrong color—O!C, (iii) wrong orientation, correct color—!OC, and (iv) both wrong responses—!O!C. For the asymmetric binding, different stimulus attributes are denoted with respect to their processing speed, slow—S, and, respectively, fast—F. See text for details.

Mandatory feature-binding would predict that random feature sampling of the different attributes of a single presented stimulus should yield accurately bound representations. In the present study, we falsify this prediction by demonstrating that different attributes of a single, focally attended stimulus may remain unbound. We developed a paradigm in which accurate responses were possible on the basis of either bound or unbound stimulus representations. If reports of different attributes rely on a bound representation, the probability of encoding accurately one attribute should co-vary with the encoding accuracy for the other. On the other hand, if unbound representations serve as the basis for responses, the encoding of the one and the other stimulus attribute should be independent. Importantly, bound or unbound encoding of different attributes would predict different probabilities of responding accurately to the one and the other attribute. Figure 1 shows alternative encoding processes, two of which assume that a subject's response is mediated by bound stimulus representations, and one of which assumes that responses to the two attributes are independent of each other. Significantly, *response* accuracy would depend both on whether or not the two attributes of the stimulus were correctly encoded and, should the encoding fail, on guessing processes which would, at least on some trials, result in correct answers.

To test the assumption that different attributes of a single, focally attended item are mandatorily bound, we looked at our results in the context of three hypotheses: on *the strong binding hypothesis*, accurately encoding one attribute (E_C_ for color and E_O_ for orientation, respectively) is not possible without accurately encoding the other attribute. Put differently, whenever a stimulus is encoded, all its attributes are encoded simultaneously and with the same speed. Consequently, reporting one attribute correctly but not the other one would be due to correct guessing for one attribute (e.g., G_O_) and an incorrect guess for the other one (e.g., !G_*C*_). Strong binding predicts no difference between tasks in which bound representations are necessary and tasks in which they are not. In other words, the strong binding predicts no binding costs, which is at variance with the available literature showing strong binding costs (e.g., Treisman, 1982, 1988; Bodelón et al., 2007, but see Holcombe and Cavanagh, 2001). Furthermore, strong binding is difficult to reconcile with reports that different attributes are processed with different speeds (Moutoussis and Zeki, 1997a,b). Taken together, the available literature raises doubt regarding the plausibility of the strong binding mechanism.

Perhaps relaxing the assumption of the strong binding model, namely that a stimulus attribute can only be encoded and processed if all other attributes were processed too, may reconcile the idea of early, perceptual binding and the evidence to the contrary. This leads to *the asymmetric binding hypothesis* which supposes that different stimulus attributes are, as with strong binding, encoded in a bound manner. The difference is that asymmetric binding permits the attribute which is encoded faster to be retrieved without having to wait for the slower attribute to finish processing. Thus, asymmetric binding would predict an asymmetry between encoding of the faster and the slower attribute: accurate encoding of the slower one would necessarily imply that the faster had been accurately encoded too. By contrast, accurately encoding the faster one would not be predictive for encoding accuracy of the slower. Asymmetric binding, unlike strong binding, would be consistent with binding costs and perceptual asynchronies: in tasks where bound representations are necessary, even though the faster attribute may already have been encoded, the response would have to wait for the slower one to be processed too. By contrast, in tasks where bound representations are not necessary, responding to the faster attribute can proceed independently of the slower one.

Finally, *independent processing* postulates that different attributes are encoded and passed to response-selection stages independently of each other. This would allow any possible combination of responses, since processing and reporting one and the other attribute are independent.

Our analyses reported below showed that the predictions of the independent model are closest to the observed data. That the independent model fits the data best, however, can have different explanations; one can postulate that stimuli are *encoded* (and retrieved) independently or that they are encoded in a bound manner but that the encoded attributes are retrieved independently (Holcombe and Cavanagh, 2008). We tested this alternative in two ways. First, the asymmetric binding model explicitly assumes that it is possible to retrieve the attribute which is processed faster before and independently from the slower attribute. Thus, comparing the goodness-of-fit between the independent and asymmetric binding models may distinguish between independent encoding and independent retrieval. Second, recent studies which investigated the representations in working memory showed that responses in a working memory task reflect a mixture of correct retrieval and guesses (Bays and Husain, 2008; Zhang and Luck, 2008). Importantly, a correct retrieval is subject to random noise so that participants on many occasions report *a similar* feature, rather than the required feature. In other words, participants more frequently make small errors (e.g., reporting pink when red was presented) relative to large ones (e.g., reporting green), yielding a non-uniform distribution of error magnitudes. When retrieval fails, participants guess randomly, predicting a uniform distribution of error magnitudes. Conceptually, several factors may contribute to the retrieval success: (i) whether or not a feature was encoded, (ii) interference between encoded features, and (iii) decay of encoded information. By presenting a single item, our study minimized potential interference. By allowing for easy verbal encoding of the presented colors and orientations (e.g., “red” or “5 o'clock”) the decay was minimized. Considering these aspects of our paradigm, precision of stimulus encoding remains the primary determinant of retrieval success. Failure to retrieve the faster attribute would predict that retrieval of the slower attribute, even a successful one, should yield nothing and participants have to guess. The guessing would result in a uniform distribution of error magnitudes for the slower attribute. By showing a strongly non-uniform distribution of error magnitudes for the slower attribute even when the error magnitude for the faster one was very high, our results render the bound encoding—independent retrieval mechanism unlikely.

## Materials and methods

Sixteen human subjects (12 females, mean age 24 years) with normal or corrected-to-normal visual acuity took part. All were shown through Ishihara tests to have normal color vision and written informed consent was obtained from all. The study conforms with the code of ethics of the World Medical Association (Declaration of Helsinki, Rickham, 1964) and was approved by an internal ethics committee.

The stimuli were presented on a 19″ CRT monitor (ViewSonic G90fB), with a screen resolution of 1024 × 768 pixels and a refresh rate of 85 Hz. Colors were selected in CIE L^*^ab color space to maintain constant luminance and saturation (CIE luminance *L* = 50, and saturation $S=\surd (a^{2}+b^{2})=10$). The hue was defined as an angle in CIE L^*^ab space: pink = 0° (CIE *a* = 40, *b* = 0), violet = 45 (CIE *a* = 28.28, *b* = −28.28), blue = 135 (−28.28, −28.28), green = 225 (−28.28, 28.28), yellow = 270 (0, 40), and red = 315 (28.28, 28.28), clockwise from pink. The colors were sampled from the whole color wheel with minimum separation of 45° resulting in eight possible colors. However, cyan (90°; CIE *a* = 0, *b* = −40), and teal color (180°; −40, 0) were not included in the experimental set because a preliminary study showed them to be difficult to distinguish from blue and green. The selected colors were then luminance-matched to the gray background (22.5 cd/m^2^, CIE L^*^ = 50, *a* = 0, *b* = 0) and the same experimental monitor was used for all participants. The six orientations were aligned as dials in an imaginary clock, from 12 o'clock (vertical or 90°) to 5 o'clock. Mask was a circular pattern consisting of a balanced mixture of all target colors created for every trial anew.

**Figure 2:**
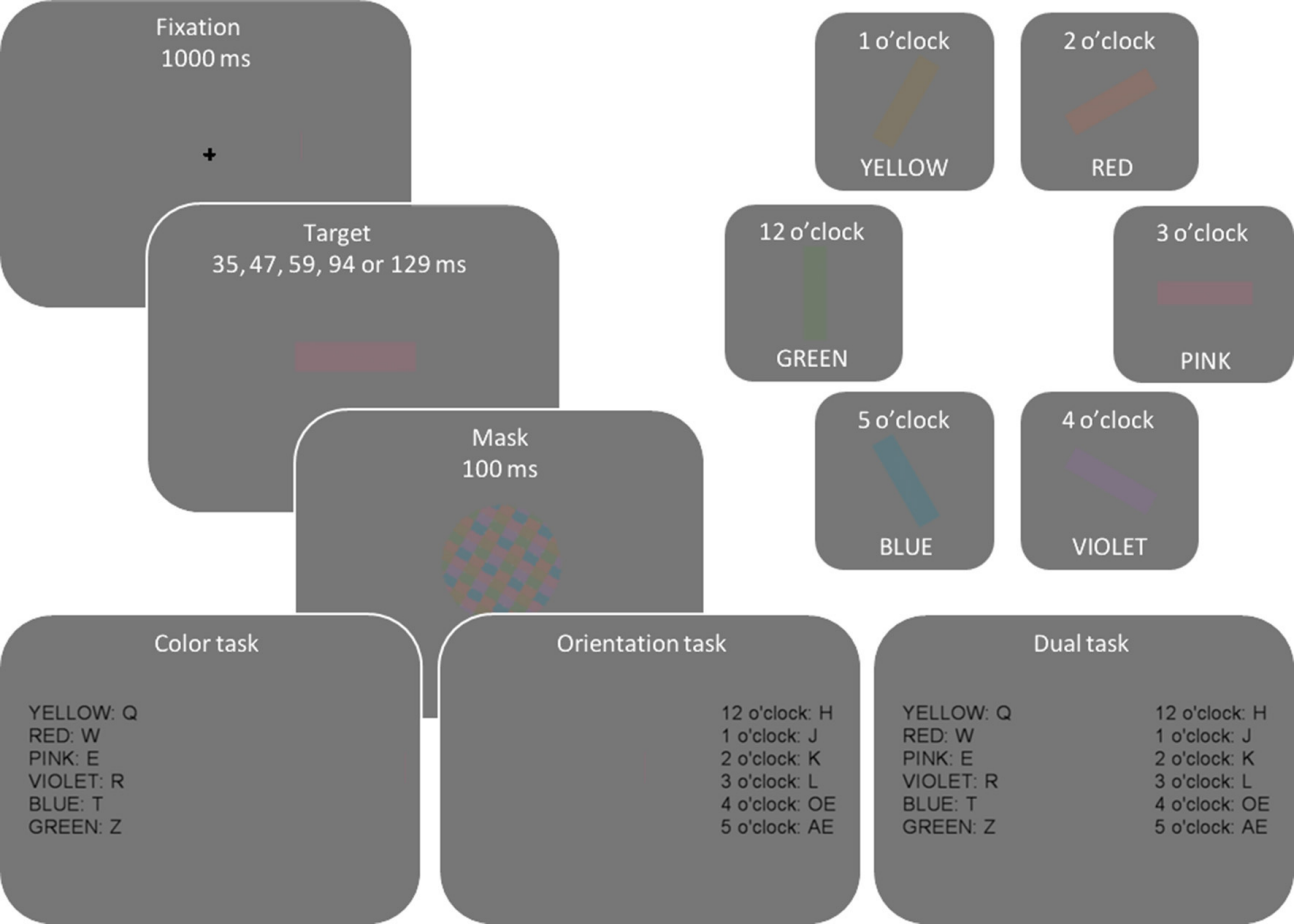
**Illustration of display sequence per trial and different color and orientation attributes**. On every trial, one color and one orientation were randomly and independently sampled from the set of depicted colors and orientations so that, across trials, participants saw any possible color-orientation combination.

Figure 2 illustrates the display sequence per trial and different colors and orientations used in the experiment. Every trial started with a fixation cross presented for 1000 ms. Next, a colored and oriented bar was randomly presented for one of five exposure durations (35, 47, 59, 94, or 129 ms), selected so that they would result in a wide performance range from random guessing to fully correct responses. Then, to eliminate residual sensory information, a mask was presented for 100 ms. To optimize response selection processes, stimulus-to-response mapping was presented at the end of each trial sequence until a response was made.

The experiment consisted of three separate sessions in which participants had to report different properties of target stimuli: in the first two (single-task) sessions they reported either the color or orientation, with the order of tasks counter-balanced across participants. In the third, dual-task session participants reported both the color and orientation of the target. A short, 5–10 min, break was introduced between sessions. The dual-task session was performed last in order to minimize errors in response selection (e.g., responding “violet” while intending to respond “red”). Such responses would be independent for color and orientation, and high frequency of these responses would have biased our data analyses toward the independent processing model.

Responses were collected via a QWERTZ keyboard. Discrimination attributes (color vs. orientation) were mapped to different hands, e.g., color-left hand, orientation-right hand. Hand-to-attribute mapping was counterbalanced across participants. The response mapping was fixed per participant and remained the same throughout the experiment. Twelve response keys, six on the upper-left part of the keyboard (q, w, e, r, t, z), and six on the middle-right keyboard section (h, j, k, l, ö, ä) were used. Color-to-key mapping was counterbalanced across participants, e.g., for pink color some participants responded by pressing key “q” and some pressed “r.” To make tasks easier, orientation-to-key mappings were kept constant such that adjacent keys served as responses to adjacent orientations, e.g., 12 o'clock = “h” and 1 o'clock =“j.”

Participants completed 10 blocks of 60 trials for each of the three experimental sessions (single task —color, single task—orientation, and dual task) yielding 1800 trials per participant. Different colors and orientations appeared equally often across trials per block (10 times) with the exact color-orientation combination randomly selected for every trial. Additionally, the stimuli were presented at different exposure durations equally often (12 times per block) yielding 120 trials per exposure duration per experimental session.

## Results

### Processing speed of color and orientation

**Figure 3:**
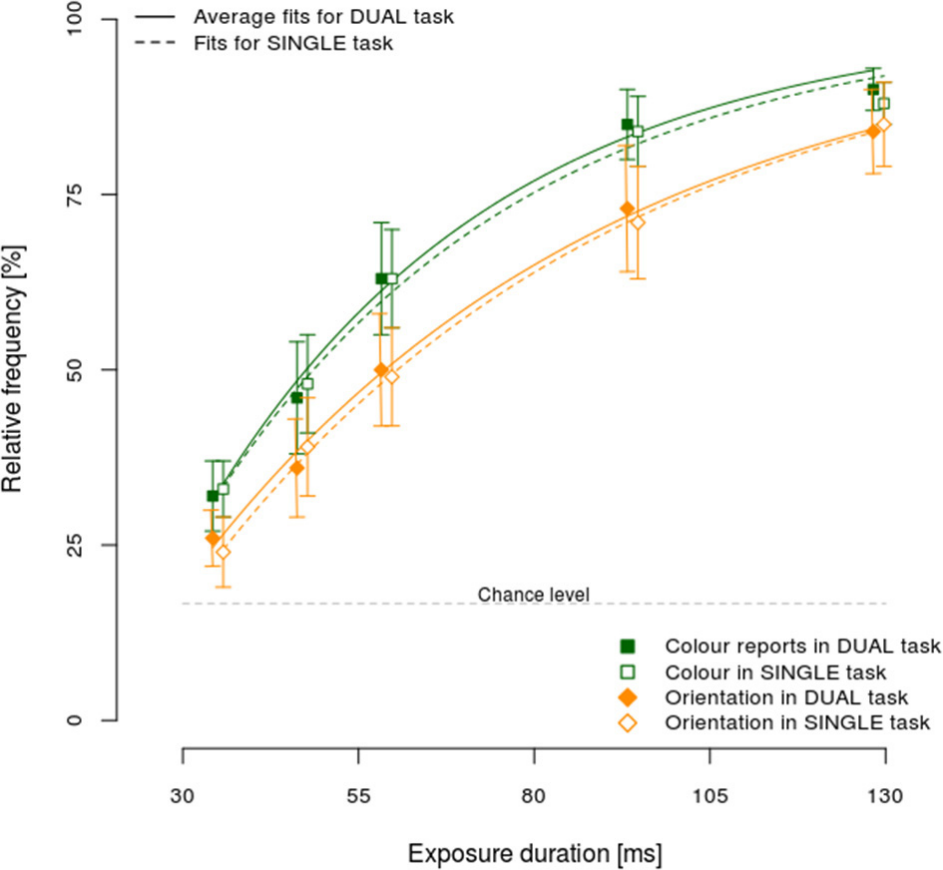
**Mean performance ±95% CI in the single and dual-task condition, separately per exposure duration and reported attribute: color (squares), and orientation (diamonds)**.

One of the postulated processing models, the asymmetric model, explicitly assumes that it should be possible to respond to the faster stimulus attribute while still processing the slower one. Consequently, an appropriate test of this model would be to establish differences in processing speeds of different stimulus attributes. Figure 3 shows the relative frequency of correct color (squares) and orientation (diamonds) responses, separately for dual (full line) and single task conditions (dashed line).

Correct color responses were more frequent than orientation ones, for all exposure durations in both the dual and single task conditions. Furthermore, the increase in response accuracy with longer exposure durations was higher for color relative to orientation, showing that color was processed faster. Additionally, the 95% confidence intervals did not include chance levels even for the shortest exposure duration, demonstrating that responses for both color and orientation were often the result of a correct encoding of the stimulus even at brief exposure durations. Finally, no substantial differences were observed between dual and single task conditions.

To test observed differences in processing speeds for color and orientation, the observed data were fitted to an exponential function separately per participant, reported attribute (color vs. orientation) and experimental condition (dual vs. single task). The following function was used *p* = 1 − *e*^*v* * (*t*−*t*0)^, where *p* denotes the proportion of correct responses, *e* the base of natural logarithm, and *t* the exposure duration. This function has been frequently used and shown to fit the data well (e.g., Bundesen, 1998). Two free parameters were fitted to the observed values, *t*_0_ and *v*, denoting, respectively, the minimum exposure duration to process stimulus correctly and the rate of increase in proportion of correct responses with an increase in exposure duration, i.e., the processing speed. The median fit across participants and conditions was very high (*R*^2^ = 0.98) indicating that the fitting procedure was successful. The mean processing speeds (i.e., *v*) for color were 2.59 and 2.89% ms^−1^ for single and dual task conditions, respectively. By contrast, the respective orientation processing speeds were 1.97 and 2.01% ms^−1^. A Two-Way repeated measures ANOVA of processing speeds across reported attribute (color/orientation) and task condition (dual/single task) showed that only the main effect of attribute was significant [*F*_(1, 15)_ = 15.46, η^2^_p_ = 0.51, *p* < 0.01], indicating significantly faster color processing (*v* = 2.74) than orientation (2.00). Neither the main effect of condition, nor its interaction with the reported attribute reached significance (both *F*s < 1.33, both *p* > 0.27). Analogous analyses of *t*_0_ showed no significant main effects or interaction (all *F*s < 1.46, all *p*s > 0.25).

Taken together, the processing speed analyses showed color to be processed faster than orientation, suggesting that our paradigm provided a good test of *asymmetric binding*. Furthermore, slower orientation processing suggests that processing of orientation relied on color contrast signals, indicating that the stimulus colors were successfully luminance-matched to the background color. Additionally, color was responded to more accurately despite the fact that stimulus-response mapping was easier for orientation where consecutive tilts (e.g., 30° and 60°) were mapped to neighboring fingers in contrast to color where no such contingencies were present. This indicates that differences in stimulus-to-response mapping did not influence processing of different stimulus attributes. Finally, the absence of a task-condition effect suggests that stimuli were processed in a comparable way both in the single and dual task condition.

### Multinomial modeling

Data from the dual-task session were used to compute parameters of different theoretical models assuming: (i) strong binding, (ii) two variants of asymmetric binding, one assuming faster color, the other assuming faster orientation processing, and (iii) independent processing. The data from the single-task sessions were used as an independent test-bed for assessing fits of different models. The responses in the dual task condition were first categorized into four types: (a) correct for both color and orientation; (b) correct for color, incorrect for orientation, (c) correct for orientation, incorrect for color, and (d) incorrect for both. Then, the relative frequencies of each response type per participant were determined and fitted to different models. Derivations of the model predictions are given in Supplementary Material. The models assume that every response is determined by the probability of perceiving a feature correctly. For the strong binding model, a single parameter (*z*) determines whether or not both attributes were correctly processed. For the asymmetric binding and independent models *two* parameters are needed, one for orientation (*x*) and one for color (*y*). The difference between asymmetric binding and independent processing is that, for the former, processing the slower attribute can only be as efficient or less efficient than processing of the faster attribute, i.e., the two parameters are not independent. By contrast, for the independent model, the processing of one and the other attribute are independent.

If the encoding of a feature failed [in (1 -*x*) and (1 -*y*) cases for orientation and color, respectively], participants were instructed to guess. With six possible colors and orientations, the probability of guessing correctly (*f* = 1/6) and of guessing incorrectly (1 − *f* = 5/6) was the same for color and orientation. The *x, y*, and *z* parameters were computed (Riefer and Batchelder, 1988) for each model, exposure duration and participant, together with the predicted values of each response type. Further, the very same *x, y*, and *z* parameters were used to predict values observed in the single-task condition. The equations used to predict the performance in the single-task are given in Supplementary Material.

**Figure 4:**
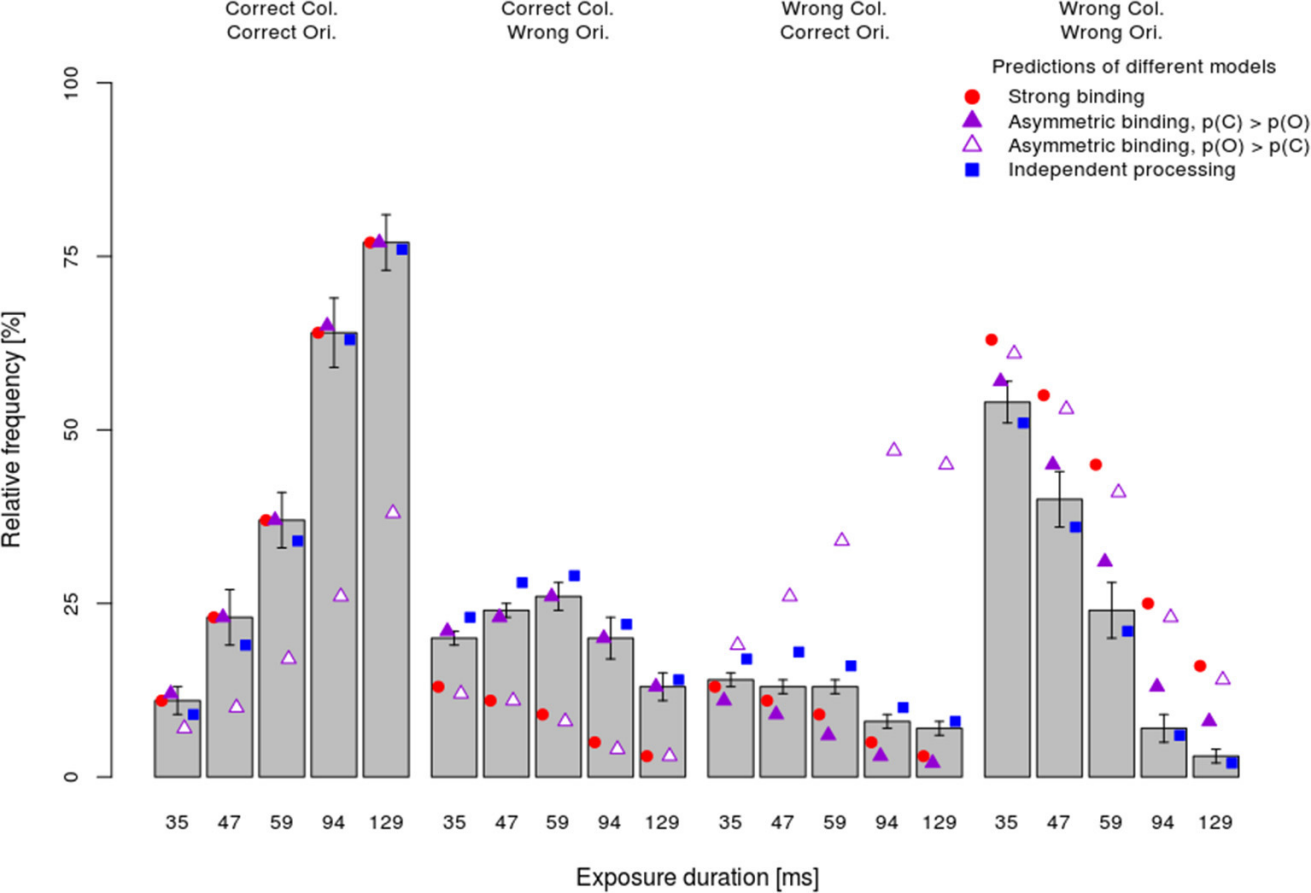
**Mean performance ±1 s.e.m. in the dual-task condition separately per response type (fully correct answers, partially correct, and fully incorrect) and exposure duration together with the predictions of different models**.

The mean *observed* frequency of different response types per stimulus exposure duration (bars) in the dual-task condition are shown in Figure 4. Consistent with the analyses of the processing speed per attribute, which showed faster color processing, the asymmetric binding model assuming faster orientation processing predicted the observed frequency very poorly (empty triangles). In particular, this model grossly overestimated the frequency of wrong color—correct orientation responses and underestimated the frequency of correct color—correct orientation responses. By contrast, other models (strong binding, asymmetric binding assuming faster color processing and independent processing) predicted well the relative frequency of fully correct responses. The strong binding model, however, systematically underestimated the frequencies of partially correct answers and overestimated the frequency of completely wrong answers. The asymmetric binding assuming faster color processing predicted well the frequencies of correct color and wrong orientation responses. However, this model underestimated the frequency of correct orientation—wrong color responses and overestimated the frequency of both wrong answers. Finally, the independent model did not show systematic deviations from the observed frequencies.

To assess how well different models fit the data, the difference between predicted and observed relative frequencies per participant was tested by Fisher's exact test. This test, rather than the more common Chi-square test, was used because for several participants with very good performance at long exposure durations, observed frequencies of completely wrong answers were very low. Table 1 shows for how many participants each of the models fitted well, i.e., for how many participants the difference between the observed and predicted frequencies failed to reach significance (p_Fisher_ > 0.05). The most striking finding is that the strong binding model, as well as asymmetric binding assuming faster orientation processing, predicted observed frequencies well for none of participants. By contrast, asymmetric binding assuming faster color processing predicted well the observed data for 10 out of 16 participants. Finally, the independent model fitted well for all participants. Binomial tests of the number of participants for which the model fitted well actually showed that the strong binding and asymmetric binding assuming faster orientation processing performed worse than expected by chance, asymmetric binding assuming faster color was at the chance level, while the independent model fitted the observed data well for more participants than expected by chance.

**Table 1:** **Number of participants (N) for which a model fit well (P_Fisher_ > 0.05) and Bayesian information criterion (BIC)**.

	**Dual task**		**Single task**	
**Model**	***N***	***BIC***	***N***	***BIC***
Strong binding	0^†^	24,081	15^‡^	13,722
Asymmetric binding				
Color faster than orientation	10	21,876	16^‡^	11,393
Orientation faster than color	0^†^	28,299	11^‡^	13,195
Independent	16^‡^	21,472	16^‡^	10,550
Independent > Strong binding	16^‡^		10	
Independent > Asymmetric, faster color	13^‡^		9	
Independent > Asymmetric, faster ori.	16^‡^		13^‡^	

*The BICs were computed on the basis of performance and parameters averaged across participants. Greater N and smaller BIC indicate better model fits*.

† *Binomial p_N_ < chance*.

‡ *Binomial p_N_ > chance*.

To compare different models more directly, the number of participants for which the independent model fitted better than the strong and the asymmetric binding models, indexed by the differences in p_Fisher_ for respective models, was computed. As shown in Table 1, the independent model fitted the data significantly better than both the strong binding and the asymmetric binding models. However, the better fits of the independent model might be trivial, simply because of a greater number of free parameters (two, *x* and *y*, for five exposure duration, i.e., 10 in total). To assess goodness-of-fit for different models independently of the number of free parameters, Bayesian Information Criterion (BIC, see Table 1), describing how much the predicted values deviate from the observed values, was computed as described in Gomez et al. (2007). Since several participants performed so well at long exposure durations that both the predicted and the observed frequencies were zero, it was not possible to compute individual BICs. We therefore used the predicted and observed frequencies, averaged across participants. However, previous studies have shown that BICs computed over averaged values correspond very well to the mean of individually computed BICs (Gomez et al., 2007), justifying this way of computing group fits. Furthermore, the number of free parameters for the independent model was set to ten, while the number of free parameters for all variants of the binding model was set to five^1^. Table 1 shows BICs for different models. As can be seen, smaller BIC was observed for the independent model relative to all other models, indicating better fits for the independent model even after penalizing for the number of free parameters.

**Figure 5:**
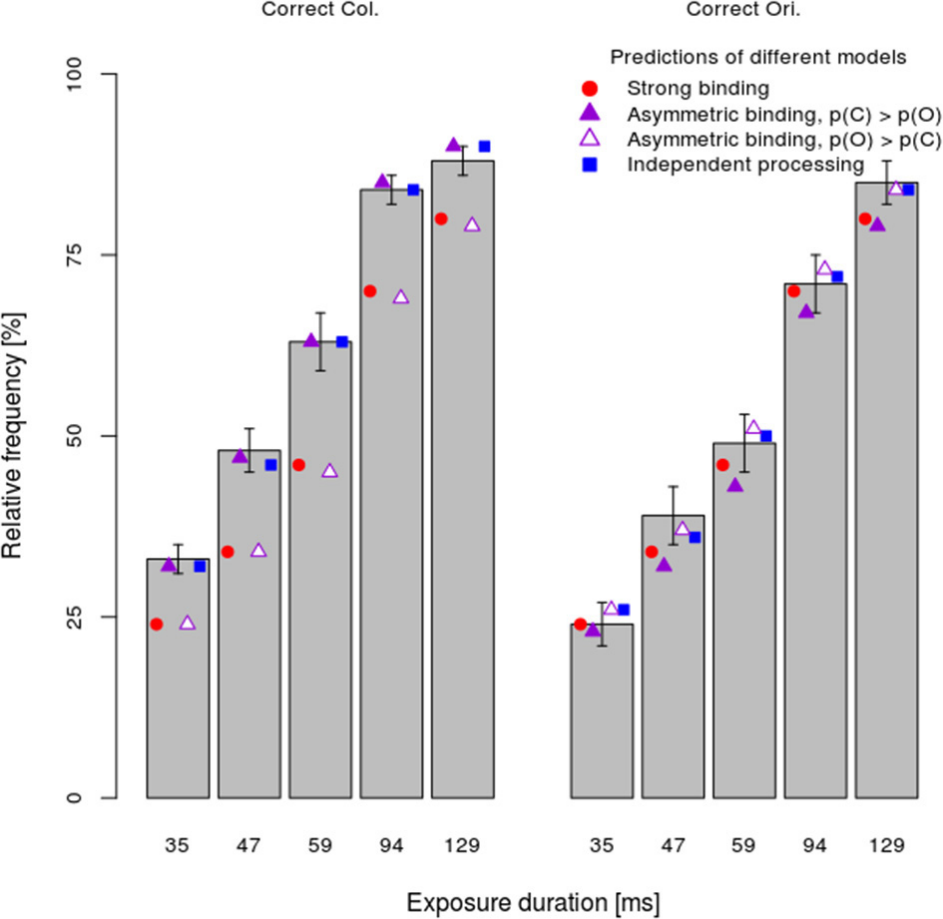
**Mean performance ±1 s.e.m. in the single-task condition separately per task (color vs. orientation discrimination) and exposure duration**. Conventions as in Figure 4.

Next, the *x, y*, and *z*-values computed for the dual task condition were used to predict relative frequencies of responses in the single task as well. Figure 5 shows the mean relative frequency of correct color and orientation responses per exposure duration (bars), together with predictions of the strong binding, different variants of the asymmetric binding and the independent models. As both Figure 5 and Table 1 show, all models fitted observed values well for the majority of participants, although computing model parameters was based on data from a different experimental condition (dual task). Binomial tests comparing the number of participants for which the independent model fits better than other models showed significant differences only between the independent model and the asymmetric binding model, assuming faster orientation processing. However, inspection of BICs showed that the independent model fits the results better than either the strong binding or the asymmetric binding assuming faster color processing.

### Error distributions

To investigate whether *encoding* or *retrieval* of different attributes takes place independently, analyses of error distributions were performed. As discussed earlier, the retrieval failures in our paradigm were primarily determined by the encoding failures. Consequently, failing to retrieve the faster-processed attribute predicts that retrieving of the slower attribute will fail too. Put differently, when the error magnitude for the faster attribute is high (reflecting guessing) a uniform distribution of error magnitudes for the slower attribute (reflecting guessing, too) is expected.

**Figure 6:**
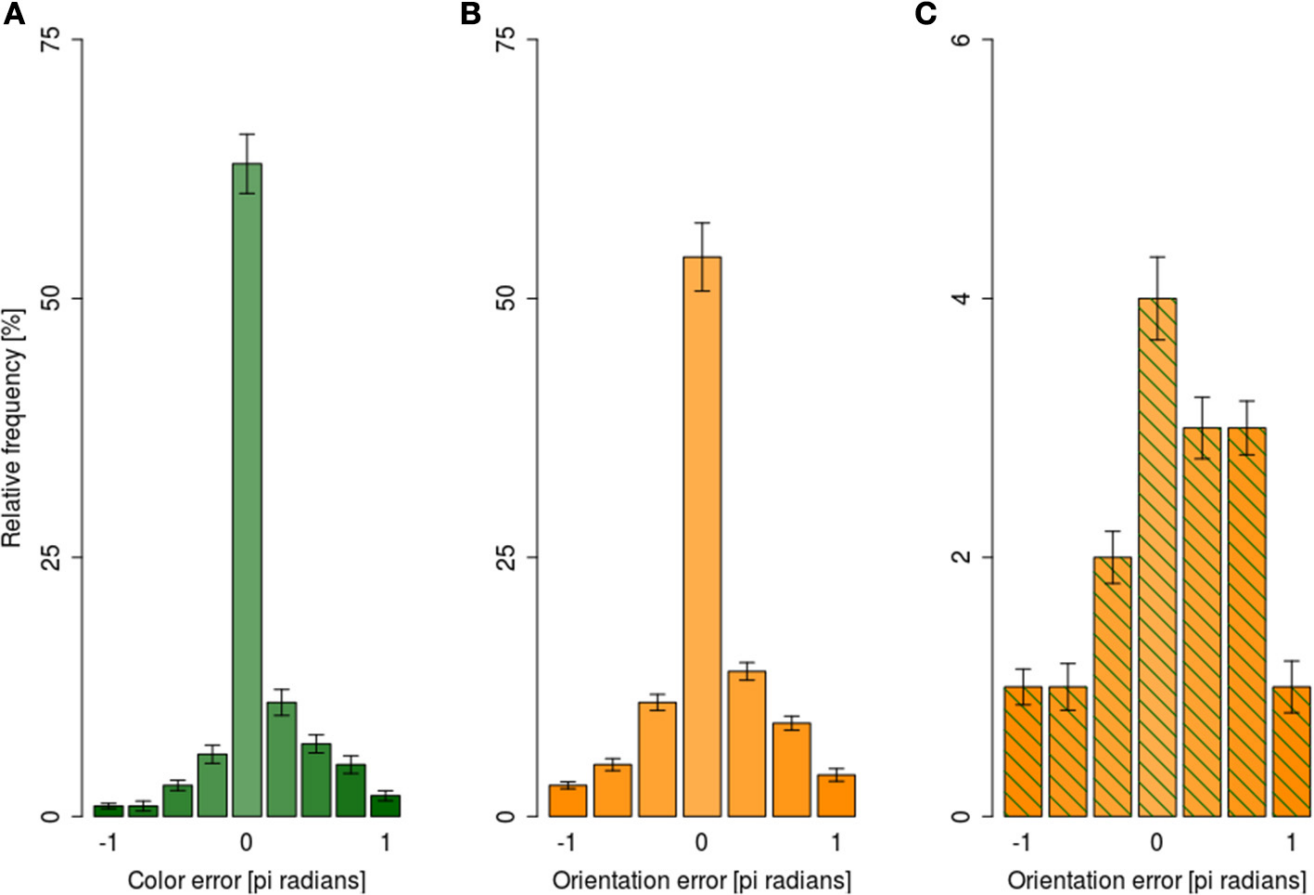
**Distribution of mean error magnitude ±1 s.e.m. in the dual-task condition collapsed across exposure durations. (A)** For color responses irrespective of the orientation error magnitude, **(B)** for orientation responses irrespective of color response magnitude, **(C)** for orientation responses when the color error magnitude was beyond 95% CI.

For analyses of the error magnitudes, each response was encoded in terms of how different it was from the correct responses yielding, e.g., 0° difference for a fully correct response and, e.g., 180° for a fully incorrect response. The angular differences were expressed as pi radians. Error magnitudes were expressed as a signed difference between the reported and presented feature. Since our responses were categorical, the distribution of error magnitudes was categorical as well: (i) there were nine possible error magnitudes for color reports, and (ii) seven possible error magnitudes for orientation responses. Figure 6A shows the distribution of error magnitudes for color independently of orientation reports, while Figure 6B shows analogous distribution for orientation collapsed across all exposure durations.

Following the suggestions of Fougnie et al. (2013) we considered color responses as guesses when they were outside of 95% confidence interval for the mean of color response distribution. Figure 6C shows the distribution of orientation error magnitudes when the response to color was a guess; the distribution was markedly non-uniform, with small errors being more likely than large ones.

To test the uniformity of error magnitude distributions shown in Figure 6, log-linear analyses of variance were performed separately for color distribution (Figure 6A), orientation distribution (Figure 6B), and orientation distribution when color responses were guesses (Figure 6C). All three analyses showed that error distributions were non-uniform, as indexed by a significant main effect of error magnitude in all three analyses (all *G*^2^ > 313, all *p*s < 0.01). Taken together, the analyses of error magnitudes showed that participants were able to retrieve the slower attribute even when the retrieval of the faster attribute failed. Should the assumption that the retrieval failures in our paradigm are due to encoding failures hold, the ability to retrieve the slower attribute when the encoding of the faster attribute failed would suggest independent encoding of different attributes of the focally attended item.

## Discussion

The present study showed higher percentages of correct color reports relative to orientation, in both the single- and dual-task conditions across all subjects and exposure durations. This is consistent with previous findings showing color to be processed faster than other visual features. Further analyses showed that the hypothesis based on the assumption that color and form are processed independently fits the observed results better than the strong or the asymmetric binding models. These results lead us to suggest that binding does not necessarily occur through interaction between specialized visual areas but is post-perceptual in nature.

Bays and colleagues, using a similar paradigm to address a different question, recently reported results suggesting that after long exposure durations different attributes of a single presented stimulus are processed in a bound manner (Bays et al., 2011). Importantly, long exposure durations most likely resulted in a very precise processing of both attributes. This may yield *an appearance* of bound representations (for a similar idea related to storage of different stimulus attributes see Fougnie et al., 2013). By using several stimulus exposure durations, our study varied how effectively a stimulus could have been encoded, allowing us to test whether or not bound processing of different attributes of a single, focally attended item still holds even when color and orientation are not fully processed.

Our results are consistent with evidence which shows that, in areas V1, V2, and V4 of macaque monkey cortex cells that are wavelength or color selective are not orientation selective (Livingstone and Hubel, 1984, 1988; Hubel and Livingstone, 1985), or have broader orientation tuning curves than orientation selective cells proper (Zeki, 1983; Economides et al., 2011; Tong et al., 2012). By contrast, narrowly tuned orientation selective cells in V1, V2, V3, and V3A are not wavelength selective (Shipp and Zeki, 1985; Felleman and Van Essen, 1987; Livingstone and Hubel, 1988). Some studies have, however, reported heavy concentrations of cells that are selective for both orientation and wavelength (Gegenfurtner et al., 1996; Johnson et al., 2001). Such double-duty cells, assuming them to exist in the human brain, would not seem to be potent enough to manifest their effects perceptually, either in experiments on perceptual asynchrony, which demonstrate that color is perceived before orientation or in the experiment reported here. What their function may be remains conjectural, and one would have to account for how their physiology reflects the perceptual realities reported here and elsewhere, especially to learn whether they respond to orientation and to wavelength with the same latency. Our results are therefore more consistent with physiological evidence which shows a separation between narrowly tuned orientation-selective but wavelength unselective cells and un-oriented wavelength-selective cells. Given the absence of a consensus about how binding occurs in the functionally specialized visual areas, we are led to propose the more radical idea that binding does not occur through physiological interactions in and between visual areas or, if it does, the effects are not reflected perceptually in our study.

The evidence that bound representations are not a necessary product of perception nevertheless raises the question of where and how different attributes of the visual world are bound together to provide a unified/bound perception as supported by evidence that humans perform very well in tasks requiring bound stimulus representations (Treisman, 1982; Holcombe and Cavanagh, 2001; Bodelón et al., 2007). A plausible explanation would be that binding occurs following allocation of focal attention, along the lines proposed in the FIT. However, while the FIT implies that all focally attended stimuli would be bound, the present study demonstrates that focal attention is not a sufficient condition for binding to occur.

**Figure 7:**
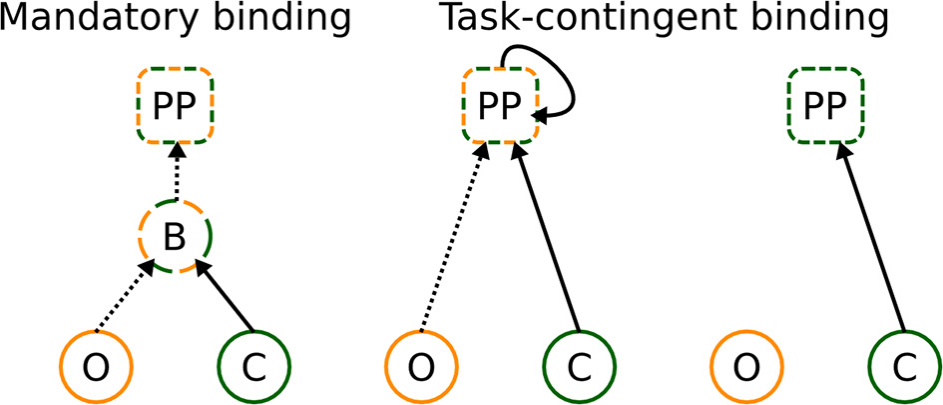
**Classical and novel explanation of binding**. It can occur in a mandatory way at perceptual stages, and already bound representations are transferred to later stages—left figure. Alternatively, binding can occur at post-perceptual stages, under conditions that bound representations are informative for solving the task—central figure. If bound representations are not required for solving the task, no binding takes place—right figure. The letters denote perceptual processing of color (C) and orientation (O), binding (B) of color and orientation, and post-perceptual (PP) stages, respectively. Dashed and full lines denote slow and fast processing speeds, respectively.

To resolve these inconsistencies, we propose that binding takes place at post-perceptual (PP) processing stages under conditions that the bound representation is helpful for solving the task (see Figure 7). Since different visual attributes are processed at different speeds, the binding processes, when taking place at all, must rely on the maintenance of the first analyzed attribute (color in the present experiment) for the period until the second attribute has been analyzed as well (indicated by a recurrent activation at PP stages, Figure 7, middle panel). In other words, the PP binding relies, to an extent, on memory processes and probably occurs in locations outside visual areas, most likely in the hippocampus, where temporal discontinuities may be bridged (Staresina and Davachi, 2009) and/or pre-frontal cortex, where integration and maintenance of several stimulus properties may occur (Freedman et al., 2001).

Although it may not appear so, the task-contingent binding mechanism is compatible with evidence of mandatory processing of task-irrelevant attributes of the attended stimuli (Valdes-Sosa et al., 2000; Snyder and Foxe, 2012; Snyder et al., 2012). In such experiments, bound representations would be of value to minimize the interference which may occur when two or more stimuli are presented. A study investigating binding of color (task-relevant) and motion (task-irrelevant) attributes showed much weaker binding indices when the two moving surfaces were spatially separated than when they overlapped (Experiment 2, Valdes-Sosa et al., 2000), although one would have expected no differences between conditions on the assumption of mandatory processing of task-irrelevant attributes. Furthermore, whether or not the motion and color were bound depended critically on experience with the stimulus material. When participants were initially presented with stationary stimuli, no evidence of color-motion binding was observed in the later, experimental phase (Experiment 4, Valdes-Sosa et al., 2000). These two findings suggest that binding task-relevant and irrelevant attributes of the attended object is not an automatic process but rather occurs because it helps solving the task. The evidence that effects of task-irrelevant attributes depend on practice supports our proposal of the task-contingent binding mechanism, which would predict that binding between different features occurs only when different features are (or have recently been) relevant, which further implies reliance on memory processes.

Finally, task-contingent binding, as described here, is relatively agnostic to the role of focal attention for binding. While the present study demonstrates that attention is not sufficient, an open question for further studies is whether or not focal attention is necessary for binding. What we propose is that neural correlates of binding should involve areas beyond sensory cortex and that memory, rather than perception, plays an important role in binding processes.

### Conflict of interest statement

The authors declare that the research was conducted in the absence of any commercial or financial relationships that could be construed as a potential conflict of interest.

## Footnotes

^1^Although the asymmetric binding model uses two parameters, *x* and *y*, these two are not completely independent, i.e., the parameter for the slower processed attribute can only be as high as the parameter for the faster attribute. Since it was not possible for us to express numerically the number of free parameters for the asymmetric model (i.e., it would have been somewhere between one and two), we computed BIC for this model in a way that should yield the best possible fit by setting the number of free parameters for the asymmetric model to be one per exposure duration, or five in total.
