# Supplementary material for: Non-binding relationship between visual features
Source: Front Hum Neurosci. 2014 Oct 8;8:749. doi: 10.3389/fnhum.2014.00749 (PMC4189329; doi:10.3389/fnhum.2014.00749)
Supplement: Supplementary file 1 [file DataSheet1.DOC]

# Appendix 1

*Table S1.* Possible response types for colour and orientation resulting from (i) correct feature encoding (C and O), (ii) correct guesses when encoding failed (C_g_ and O_g_) and (iii) incorrect guesses (!C and !O) along with the associated response probabilities, on the assumptions of the strong and weak binding, and the independent models: *x* and *y* denote probability of encoding correctly the orientation and colour attributes, respectively, and *fx* and *fy* denote probability of the guessing correctly the orientation and colour attributes, respectively. Notice that the strong binding model has a single parameter (*z*), that of encoding correctly all stimulus’ attributes: this parameter is equal to the probability of encoding correctly the *slower* attribute, or the orientation (*x*) in the present study. For the asymmetric binding, the *x* denotes the probability of encoding correctly the *slower* attribute (S), and *y* the probability of encoding correctly the *faster* attribute (F).

| Orientation | | Colour | | | |
| --- | --- | --- | --- | --- | --- |
| Response | Probability | Response | Probability | | |
|  |  |  | Strong binding* | Asymmetric binding | Independent |
| O/S | *x* | C/F | 1 | 1 | *y* |
|  |  | C_g_/F_g_ | 0 | 0 | (1-*y*)*f_y_* |
|  |  | !C/!F | 0 | 0 | (1-*y*)(1-*f_y_*) |
| O_g_/S_g_ | (1-*x*)*f_x_* | C/F | 0 | *y* | *y* |
|  |  | C_g_/F_g_ | *f_y_* | (1-*y*)*f_y_* | (1-*y*)*f_y_* |
|  |  | !C/!F | (1-*f_y_*) | (1-*y*)(1-*f_y_*) | (1-*y*)(1-*f_y_*) |
| !O/!S | (1-*x*)(1-*f_x_*) | C/F | 0 | *y* | *y* |
|  |  | C_g_/F_g_ | *f_y_* | (1-*y*)*f_y_* | (1-*y*)*f_y_* |
|  |  | !C/!F | (1-*f_y_*) | (1-*y*)(1-*f_y_*) | (1-*y*)(1-*f_y_*) |

* For the strong binding model *x* = *z*

The probability of different *observed* response types (OC, O!C, !OC, and !O!C) is a combination of probabilities of encoding stimulus attributes correctly (O and C) and, if encoding failed, probabilities of correct and incorrect guesses (O_g_, C_g_, !O and !C):

1. *p_OC_ = p_O_p_C_ + p_O_p_Cg_ + p_Og_p_C_ + p_Og_p_Cg_*
2. *p_O!C_ = p_O_p_!C_ + p_Og_p_!C_*
3. *p_!OC_ = p_!O_p_C_ + p_!O_p_Cg_*
4. *p_!O!C_ = p_!O_p_!C_*

Combining the probabilities of responses predicted on the basis of different models (Table 1) with the equations 1-4 results in three systems of four equations, one for each of the three models. Using procedure described in Riefer and Batchefelder (1988), these systems were solved for parameters x, y, and z, resulting in the following solutions, with N denoting the number of trials, i.e., the frequency of a response type:

1. for the strong binding model

*z = [N_OC_ – (N_OC_ + N_O!C_ + N_!OC_ + N_!O!C_)f_x_f_y_]/[(N_OC_ + N_O!C_ + N_!OC_ + N_!O!C_)(1 – f_x_f_y_)]*

1. for the asymmetric binding model

*x = [N_OC_ – (N_OC_ + N_!OC_)f_x_]/ [(N_OC_ + N_O!C_ + N_!OC_ + N_!O!C_)(1 – f_x_)]*

*y = [N_!OC_ + (N_O!C_ + N_!O!C_)f_x_f_y_ – (N_O!C_ + N_!OC_ + N_!O!C_)f_y_]/[(N_O!C_ + N_!OC_ + N_!O!C_ – N_O!C_f_x_ – N_!O!C_f_x_)(1 - f_y_)]*

1. for the independent model

*x = [(N_OC_ + N_O!C_) – (N_OC_ + N_O!C_ + N_!OC_ + N_!O!C_)f_x_]/[(N_OC_ + N_O!C_ + N_!OC_ + N_!O!C_)(1 – f_x_)]*

*y = [(N_OC_ + N_!OC_) – (N_OC_ + N_O!C_ + N_!OC_ + N_!O!C_)f_y_]/[(N_OC_ + N_O!C_ + N_!OC_ + N_!O!C_)(1 – f_y_)]*

# Appendix 2

The relative frequency of correct colour and orientation responses in the single task condition was predicted as the sum correct responses to one attribute, e.g., colour, in cases when the other response was correct and incorrect:

1. *p_O_ = p_OC_ + p_O!C_*
2. *p_C_ = p_OC_ + p_!OC_*

Replacing *p_OC_, p_O!C_,* and *p_!OC_* with equations 1 – 3 from Appendix 1, and solving them for x and y parameters yielded following solutions for different processing models:

1. for the strong binding and independent processing models:

*p_O_ = x + (1 - x)f_x_*

*p_C_ = y + (1 - y)f_y_*

1. for the asymmetric binding model:

*p_S_ = x + (1 - x)f_x_*

*p_F_ = x + (1 - x)[y + (1 - y)f_y_]*

# Appendix 3

Several recent studies (Bays & Husain, 2008; Bays et al., 2009; Bays et al., 2011) demonstrated that some incorrect responses may actually be considered as correct but imprecise responses, e.g., reporting pink when red was presented. Consequently, computing response accuracy solely on the basis of whether or not a response was correct and disregarding the response precision would underestimate the frequency of accurate and overestimate the frequency of inaccurate responses. To estimate whether or not the results of the present study were specific for the method of computing response accuracy, we re-coded incorrect colour and orientation reports with a small error magnitude as correct responses. Then, we recomputed the relative frequencies of different response types and fitted different multinomial models to these frequencies. Figures S1 and S2 show the observed and predicted frequencies of different response types separately per exposure duration for the dual and single task condition, respectively.

*
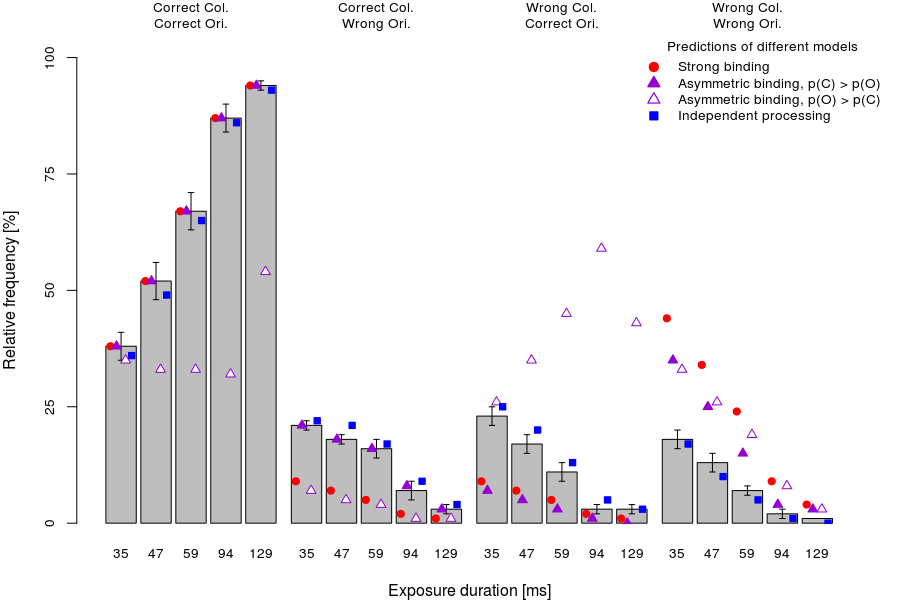
*

**Figure S1**. Mean performance ± 1 SEM in the dual-task condition separately per response type (fully correct answers, partially correct, and fully incorrect) and exposure duration. Inaccurate responses with a minimal error magnitude were considered as accurate, noisy responses. Conventions as in Figure 4.

*
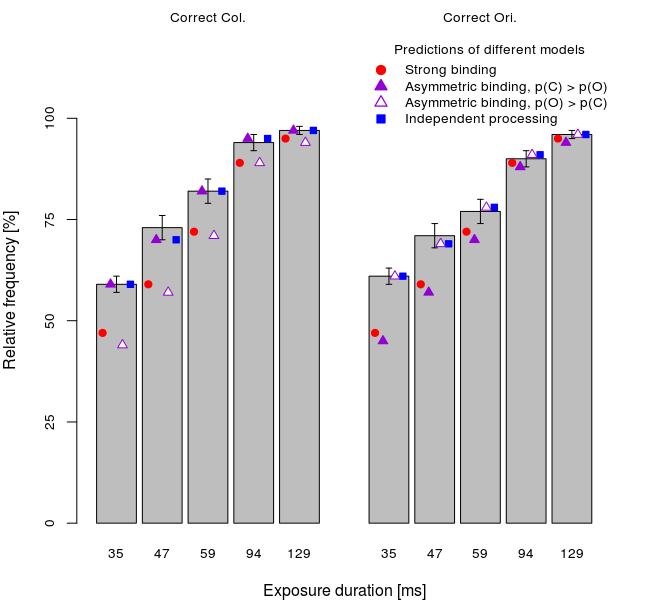
*

**Figure S2**. Mean performance ± 1 SEM in the single-task condition separately per task (colour vs. orientation discrimination) and exposure duration together with the predictions of different models. Inaccurate responses with a minimal error magnitude were considered as accurate, noisy responses. Conventions as in Figure 4.

As expected, the new computations yielded higher frequencies of correct responses and lower frequencies of incorrect responses relative to the original computations shown in Figures 4 and 5. Critically, predictions of the strong and asymmetric binding models showed systematic deviations from the computed relative frequencies similar to the deviations observed in the original analyses. In particular, both variants of binding models underestimated the frequency of partially correct reports, e.g., correct colour and incorrect orientation, as well as overestimated the frequency of completely incorrect responses, e.g., both colour and orientation incorrect.

Table S2 shows the goodness-of-fit for different models, computed in the same way as in the original analyses. For the dual-task condition, both the strong and asymmetric binding models failed to predict observed values for most of the participants. By contrast, the independent model predicted well the observed values for every participant. For the single-task condition, all models predicted the observed values well. Importantly, the goodness-of-fit as indicated by the BIC values was best for the independent model.

***Table S2****.* Number of participants (N) for which a model fit well (P_Fisher_ > .05) and Bayesian information criterion (BIC). The BICs were computed on the basis of performance and parameters averaged across participants. Greater N and smaller BIC indicate better model fits.

|  | **Dual task** | | **Single task** | |
| --- | --- | --- | --- | --- |
| **Model** | **N** | **BIC** | **N** | **BIC** |
| Strong binding | 0^†^ | 19,599 | 16^‡^ | 9,655 |
| Asymmetric binding |  |  |  |  |
| Colour faster than orientation | 2^†^ | NA* | 16^‡^ | 8,295 |
| Orientation faster than colour | 0^†^ | 25,227 | 16^†^ | 8,595 |
| Independent | 16^‡^ | NA* | 16^‡^ | 6,598 |
| Independent > Strong binding | 16^‡^ |  | 13^‡^ |  |
| Independent > Asymmetric, faster colour | 16^‡^ |  | 14^‡^ |  |
| Independent > Asymmetric, faster ori. | 16^‡^ |  | 13^‡^ |  |

^†^Binomial p_N < chance_

^‡^Binomial p_N > chance_

* Computing BICs was impossible since predicted values for !O!C response type at long exposure durations were zero.

Taken together, analyses of how well different models predicted frequency of different response types computed in a modified way again showed superiority of the independent model over the other two models. This suggests that the best fit for the independent model was not simply a consequence of computation methods.
